# Low Phase Angle Values Are Associated with Malnutrition according to the Global Leadership Initiative on Malnutrition Criteria in Kidney Transplant Candidates: Preliminary Assessment of Diagnostic Accuracy in the FRAILMar Study

**DOI:** 10.3390/nu15051084

**Published:** 2023-02-21

**Authors:** Elena Muñoz-Redondo, Andrea Morgado-Pérez, María-José Pérez-Sáez, Anna Faura, Dolores Sánchez-Rodríguez, Marta Tejero-Sánchez, Delky Meza-Valderrama, María Dolors Muns, Julio Pascual, Ester Marco

**Affiliations:** 1 Physical Medicine and Rehabilitation Department, Parc de Salut Mar (Hospital del Mar–Hospital de l’Esperança), 08003 Barcelona, Spain; 2 Rehabilitation Research Group, Hospital del Mar Medical Research Group, 08003 Barcelona, Spain; 3 PhD Program in Medicine, Department of Medicine, Universitat Autònoma de Barcelona, 08035 Barcelona, Spain; 4 Nephrology Department, Hospital del Mar, 08003 Barcelona, Spain; 5 Kidney Disease Research Group, Hospital del Mar Medical Research Group, 08003 Barcelona, Spain; 6 Faculty of Health and Life Sciences, Universitat Pompeu Fabra, Dr Aiguader Building (Mar Campus), 08003 Barcelona, Spain; 7 Geriatrics Department, Brugmann University Hospital, Université Libre de Bruxelles, 1020 Brussels, Belgium; 8 Geriatrics Department, Parc de Salut Mar (Centre Fòrum), 08019 Barcelona, Spain; 9 WHO Collaborating Centre for Public Health Aspects of Musculo-Skeletal Health and Ageing, Division of Public Health, Epidemiology and Health Economics, Université of Liège, Campus Sart Tilman, Quartier Hôpital, 4000 Liège, Belgium; 10 Physical Medicine and Rehabilitation Department, National Institute of Physical Medicine and Rehabilitation (INMFRE), Diagonal a la Universidad Tecnológica de Panamá, Panama City 0819, Panama; 11 Department of Endocrinology and Nutrition, Hospital del Mar, 08003 Barcelona, Spain; 12 Nephrology Department, Hospital Universitario 12 de Octubre, 28041 Madrid, Spain

**Keywords:** phase angle, malnutrition, GLIM, muscle mass, advanced chronic kidney disease, prehabilitation

## Abstract

Malnutrition has a negative impact on patients with chronic diseases and its early identification is a priority. The primary objective of this diagnostic accuracy study was to assess the performance of the phase angle (PhA), a bioimpedance analysis (BIA)-derived parameter, for malnutrition screening using the Global Leadership Initiative for Malnutrition (GLIM) criteria as the reference standard in patients with advanced chronic kidney disease (CKD) waiting for kidney transplantation (KT); criteria associated with low PhA in this population were also analyzed. Sensitivity, specificity, accuracy, positive and negative likelihood ratios, predictive values, and area under the receiver operating characteristic curve were calculated for PhA (index test) and compared with GLIM criteria (reference standard). Of 63 patients (62.9 years old; 76.2% men), 22 (34.9%) had malnutrition. The PhA threshold with the highest accuracy was ≤4.85° (sensitivity 72.7%, specificity 65.9%, and positive and negative likelihood ratios 2.13 and 0.41, respectively). A PhA ≤ 4.85° was associated with a 3.5-fold higher malnutrition risk (OR = 3.53 (CI95% 1.0–12.1)). Considering the GLIM criteria as the reference standard, a PhA ≤ 4.85° showed only fair validity for detecting malnutrition, and thus cannot be recommended as a stand-alone screening tool in this population.

## 1. Introduction

Kidney transplant (KT) candidates usually experience limitations in physical function and exercise capacity [1], with a negative impact on functional status, quality of life, and clinical outcomes [2]. Low levels of physical activity are associated with worse results post-KT, including lower cardiorespiratory capacity, metabolic and nutritional impairment, reduction in quality of life, and increased mortality [3,4]. The loss of functional capacity is multifactorial: aging, comorbidities, nutritional disorders, and frailty, among others, play an important role [5]. The identification of the links between aging, nutritional disorders, physical function, and chronic diseases is a priority for the professional clinical nutrition community and the largest societies of clinical nutrition and metabolism, among others [6].

The assessment of body composition is a major challenge in patients with chronic kidney disease (CKD). A wide variety of methods have been used to assess muscle mass, ranging from visual signs and anthropometric measures to creatine kinetics, bioelectrical impedance, whole-body counting, neutron activation analysis (i.e., total body nitrogen), and imaging techniques such as dual energy X-ray absorptiometry (DXA), computed tomography (CT), and magnetic resonance imaging (MRI) [7]. Although imaging methods are the most precise and accurate for assessing muscle mass [8], bioimpedance techniques (i.e., bioelectrical impedance analysis (BIA) and bioelectrical impedance spectroscopy (BIS)) are more frequently used in clinical settings [7].

The phase angle (PhA) is a BIA-derived parameter associated with the size and integrity of the cell membrane. Normal PhA values are 5–7° in healthy adults, and are usually lower in women than in men, except in people aged 70 and older [9,10]. Some studies have shown differences in PhA due to race, with the highest values obtained in the African American and Hispanic populations and the lowest obtained in the Asian population [9]. The PhA tends to increase as body mass index (BMI) increases up to 35 kg/m^2^; with a higher BMI, PhA decreases [9]. A positive correlation between PhA and muscle strength has also been described in older adults [10]. A recent systematic review on the relationship of PhA and malnutrition evaluated using the subjective global assessment (SGA) reported trends of reduced PhA values in patients with malnutrition in the vast majority of the studies included. However, the review was not able to conclude that PhA could independently identify malnutrition in adults (disease-related malnutrition); moreover, the Global Leadership Initiative on Malnutrition (GLIM) criteria, the most updated operational definition of malnutrition in adults, were not applied [11].

The European Society of Clinical Nutrition and Metabolism (ESPEN) has recently launched the guidance for the assessment of the muscle mass phenotypic criterion required by GLIM for the diagnosis of malnutrition. This evidence-based and consensus-based guidance aims to promote the assessment of muscle mass as a crucial criterion in the assessment of malnutrition according to the GLIM [12]. The guidance advocated that quantitative assessment methods such as BIA be given priority for muscle mass assessment. This decision was made because BIA has been shown to be one of the most reliable, non-invasive, inexpensive, and feasible methods for muscle mass assessment and monitoring in clinical practice and research [9,12]. The guidance states that BIA is reliable as long as results for each BIA-related parameter are cautiously analyzed in the different populations. The guidance highlights the potential role of the PhA, which is gaining attention as a surrogate marker for muscle mass. Recent studies have shown that a reduced PhA might be associated with malnutrition, and might be useful for anticipating disease prognosis and mortality in healthy older people [10,13].

Under the hypothesis that the PhA could play a role in the screening for nutritional disorders, this study was aimed at assessing the performance properties of the PhA for malnutrition screening, using the GLIM criteria as the reference standard in prehabilitation patients with advanced CKD on the waiting list for KT in the FRAILMar study; the individual phenotypic criterion associated with a low PhA in this population was also analyzed.

## 2. Materials and Methods

### 2.1. Study Design

A diagnostic accuracy study was undertaken with baseline data from the FRAILMar cohort, which assessed patients with advanced CKD awaiting KT and participating in a multimodal prehabilitation program [14]. The Standards for Reporting of Diagnostic Accuracy Studies (STARD) were followed [15].

### 2.2. Setting

The setting was the Physical Medicine and Rehabilitation Department of a university hospital in Barcelona (Catalonia, Spain).

### 2.3. Participants

The FRAILMar cohort included adults with advanced CKD included on the KT deceased-donor waiting list and referred to a prehabilitation program for functional optimization. Patients with muscle diseases or injuries or who could not perform the exercise program were excluded. For the present diagnostic accuracy analysis, two additional inclusion criteria were applied: completion of the FRAILMar baseline assessment and available data on BIA-derived parameters at baseline.

### 2.4. Test Methods

The index test to screen for malnutrition, in addition to existing protocols, was the measurement of the PhA using the InBody S10^®^ multi-frequency system (Biospace, California, USA); this BIA device applies 1, 5, 50, 250, 500, and 1000 Hz frequencies. PhA was calculated at 50 Hz frequency. Patients removed any metal objects and rested supine on a stretcher for 10 min. Tactile electrodes were placed on the thumb and middle fingers of both hands and below the malleoli of both ankles. Before placing the electrodes, a wipe recommended by the manufacturer was used to improve their electrical conductivity.

The reference standard was the GLIM criteria, whereby the diagnosis of malnutrition is based on the presence of at least one etiologic and one phenotypic criterion (low body mass index, low muscle mass, and/or unintentional weight loss) [16]. The authors considered CKD as the etiologic criterion of disease burden. Muscle mass was studied with the body composition parameters provided by the BIA device (InBody S10^®^ multi-frequency system, Biospace, California, USA); fat-free mass was expressed in kg and as a percentage of the European population reference values [17], appendicular musculoskeletal mass index (AMMI) (kg/m^2^), and musculoskeletal mass (kg). Other data on body composition were collected, including body fat (kg and as percentage of body weight) [17], total body water (TBW), extracellular water (ECW), and intracellular water (ICW), all in liters (L), and the ECW/TBW ratio (ECW/TBW >0.390 indicated overhydration, and <0.360 indicated dehydration [18,19]).

Given the post hoc nature of this diagnostic accuracy analysis, the reference standard results were unavailable to the researcher who performed the index test. Similarly, the index test measurements were unavailable to the evaluators of the reference standard.

### 2.5. Other Study Variables

Muscle function: Upper limb muscle strength was measured via maximal isometric contraction of the flexor muscles of the hand (handgrip) using a digital dynamometer (Jamar Plus^®^, Nottinghamshire, UK) and standardized procedures [20] and following the Southampton protocol [21]. The patient was seated in a chair with a backrest and without armrests, with the elbow flexed at 90° and the arm slightly separated from the body. The highest value of three reproducible maneuvers (<10% variability between values) was used for analysis. Handgrip values were expressed in kg and as a percentage of the reference population [22].

Muscle quantity: Ultrasound assessment of the upper and lower limb muscle thickness were used to assess muscle quantity. For the upper limbs, the thickness of the supinator, extensor carpi brevis, extensor carpi longus, and brachioradialis muscles was assessed using muscular ultrasound. The ultrasound measurement was performed with the patient seated, the forearm resting on a table, and the hand holding a ball without exerting pressure (in order to maintain the position). The forearm was measured from the bicipital tendon to the radial styloid and the probe was placed transversely in the proximal third of the forearm. Three images were taken and the three values were averaged. The non-dialysis fistula upper arm was used for assessments. Ultrasound of the lower extremities was performed in the supine position with the legs straight. A leg measurement was taken from the greater trochanter to the superior pole of the patella and the probe was placed transversely at the midpoint. The thickness of the rectus femoris muscle of the dominant lower limb was evaluated. Several measurements were taken along the muscle belly from the inferior to superior aponeurosis. Three reproducible measurements were selected from different images and averaged. The SARCUS protocol was followed for the measurements via ultrasound [23,24].

Potential confounders were dialysis modality (hemodialysis, dialysis peritoneal, and non-dialysis) and frailty, understood as a state of increased vulnerability to health problems. Frailty was assessed using the Fried phenotype, including unintentional weight loss (>4.5 kg or >5% of body weight (via direct measurement) in previous year), reduced handgrip strength (lower 20% of reference value, adjusted for sex and body mass index), decreased energy level (self-report of exhaustion, identified by two questions from the Center for Epidemiologic Studies Depression Scale), slow walk speed (walking time/4 m in the slowest 20% by sex and height), and physical inactivity (a weighted score of kilocalories expended per week in the lowest 20%, based on the short version of the Minnesota Leisure Time Activity questionnaire) [25]. Patients with Fried scores of 3 or higher were considered to be frail [26].

### 2.6. Study Procedure

Within the baseline assessment (before beginning prehabilitation), demographic, anthropometric, and clinical data were collected and all functional tests were carried out by a trained researcher of the FRAILMar study. Another member of the research team reviewed the results and applied the GLIM criteria. Two-day assessments were conducted at the Rehabilitation Exercise Laboratory. Non-dialysis patients were scheduled at their convenience. Those receiving renal replacement therapy were assessed within 24 h post-hemodialysis, and if they were on peritoneal dialysis, they were told to attend with an empty cavity. There was no time interval for the assessment of PhA and the application of the GLIM criteria.

### 2.7. Ethics

National and international ethics recommendations for research with human beings were followed, including the Good Clinical Practice Guidelines, the Code of Ethics, and the Declaration of Helsinki with its further amendments. The data were processed in accordance with the provisions of current legislation in Spain and the General Data Protection Regulation in the European Union 2016/679 of the European Parliament and of the Council, dated 27 April 2016. All participants received written information about the trial procedures and signed the informed consent. The study protocol and the informed consent were reviewed and approved by the local Ethics Committee of the Hospital del Mar Research Institute, Barcelona, Catalonia, Spain (Nr. 019/8623/I).

### 2.8. Statistics

Categorical variables were expressed in absolute values and percentages; quantitative variables were expressed through their mean and SD. The assumption of normality was analyzed through normality plots and by using the Kolmogorov–Smirnov test corrected with the Lilliefors test. The main performance properties were calculated: sensitivity, specificity, positive predictive value (PPV), negative predictive value (NPV), accuracy index (proportion of true results for both index and reference tests), and positive and negative likelihood ratios (LR+ and LR-, respectively). The established thresholds for validity were considered as follows: sensitivity or specificity <50% (poor validity); sensitivity or specificity <80% but both values >50% (fair validity); and sensitivity and specificity >80% (good validity) [27]. LR >1 indicates that the test result is associated with malnutrition, while <1 is associated with the absence of malnutrition. LR+ >10 and LR− <0.1 indicate a relevant change in the pretest probability [28,29]. The reliability indexes of the PhA values in comparison to the GLIM criteria were studied using contingency tables. Each 2 × 2 contingency table contained one row for low or normal PhA and two columns for GLIM criteria (dichotomous variable: yes or no). The area under the receiver operating characteristic (ROC) curve was used to evaluate the accuracy of the PhA values to predict malnutrition [30]; values close to 1 were associated with better diagnostic accuracy [31]. The optimal threshold was determined by the maximum Youden index, which summarizes sensitivity and specificity and ranges from 0 to 1: a value of 0 indicates that the assessment method is useless and 1 indicates perfect sensitivity and specificity. Given the characteristics of the FRAILMar study and the existing literature on the association between low BMI and low PhA [11], a complementary analysis was run to determine the performance properties of the PhA as a screening method in the subsample of study participants with a BMI < 25 kg/m^2^.

Univariate and multivariate logistic regressions were performed to determine which of the covariates were associated with a threshold for low PhA. Results were expressed as the crude or adjusted odds ratio (OR) with its 95% confidence interval (CI), as appropriate (univariate and multivariate analysis, respectively). Multivariate analysis included the variables significant at univariate analysis, along with those that are clinically relevant. *p*-values <0.05 were considered to be statistically significant. Analysis was performed using the IBM Statistical Package for Social Sciences (SPSS version 25.0; SPSS, Inc., Chicago, IL, USA).

## 3. Results

**Figure 1:**
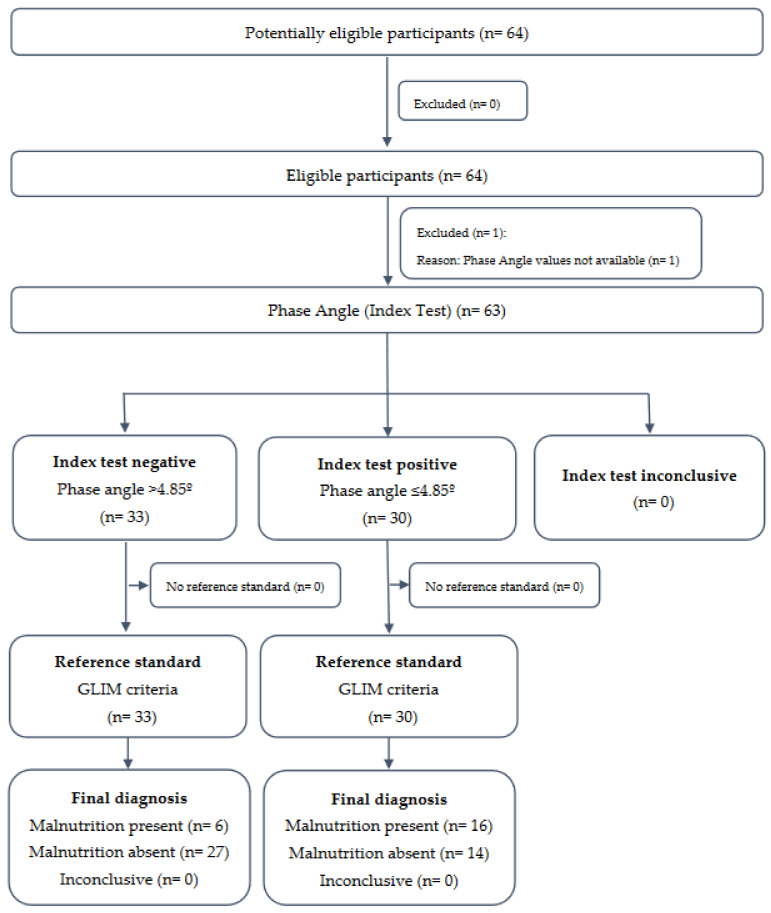
STARD diagram reporting flow of participants (*n* = 64).

From 64 patients included in the FRAILMar study, data on BIA-derived parameters were available in 63 participants (mean age 62.9 (SD 10.9) years; 76.2% men) (Figure 1). Demographic and clinical characteristics of the study participants regarding nutritional assessment, muscle size and function, and physical performance are displayed in Table 1. The prevalence of malnutrition according to the GLIM criteria was 34.9% (22 participants). The mean PhA value was 5.0 (SD 0.9); 51 patients (81%) had reduced values compared to the reference values [32].

Mean fat-free mass was within the normality range of the reference population [17]. All patients had increased percentages of body fat (mean 31.6 (SD 10.0) in men; 40.7 (SD 2.2) in women). Mean ECW/TBW was 0.397 which indicates slight edema; hydration status was normal in only 18 patients (28.6%).

**Figure 2:**
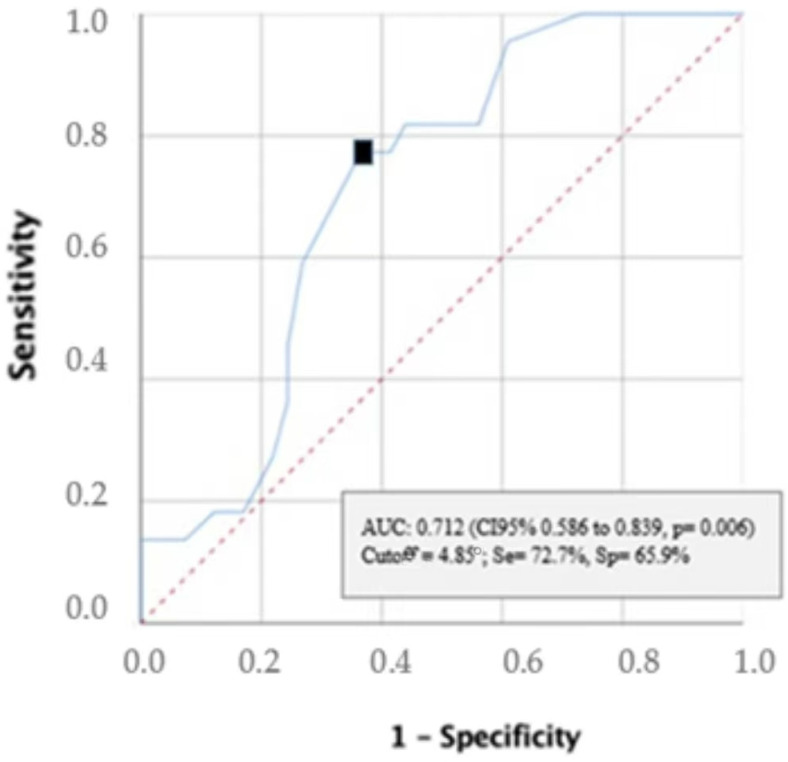
Receiver operating characteristic (ROC) curve for prediction of malnutrition based on the GLIM criteria.

**Figure 3:**
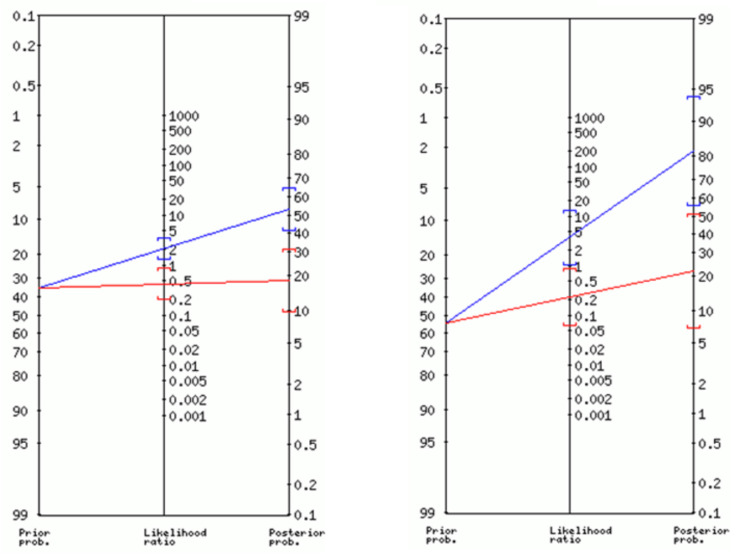
Post-test positive (blue line) and negative (red line) probabilities of malnutrition in candidates on the waiting list for kidney transplantation (**left**) and in the subgroup of subjects with BMI < 25 kg/m^2^ (**right**). Nomograms computed with the ‘Diagnostic Test Calculator’ (available at araw.mede.uic.edu).

The area under the ROC curve was 0.712 (95% CI 0.586 to 0.839, *p* = 0.006) (Figure 2). A PhA threshold of ≤4.85° was determined by the maximum Youden index, and showed an accuracy of 68.3%, sensitivity of 72.7%, and specificity of 65.9% (fair validity). A contingency table (Table 2) was used to show the frequency distribution of malnourished patients according to the PhA threshold of ≤4.85°. The area under the ROC curve (AUC) was 0.712 (95% CI 0.586–0.839) (Figure 2); this AUC indicates nearly 71.2% probability that a person with malnutrition would have a PhA ≤ 4.85°. The main performance properties of PhA are summarized in Table 3. The performance properties of PhA improved when the test was administered in the subsample of participants with a BMI < 25 kg/m^2^, as shown in Table 3. Figure 3 displays the probabilities that a patient had malnutrition after a positive or negative test for all of the study sample, and for the subgroup of subjects with a BMI < 25 kg/m^2^.

The univariate analysis according to the PhA cutoff detected significant differences in age, frailty status, and muscle thickness (Table 4). In the multivariate logistic regression analysis, the crude OR for malnutrition was 5.14 (CI 95% 1.7 to 16.1, *p* = 0.005) and the adjusted OR was 3.8 (CI 95% 1.1 to 13.8, *p* = 042) after adjusting for age, frailty status, and handgrip strength (Table 5). There were no missing data. No adverse events derived from the administration of the various tests were reported.

## 4. Discussion

This diagnostic accuracy study assessed the PhA values and their potential usefulness as a biomarker and screening tool of malnutrition in patients with advanced CKD waiting for KT in the baseline assessment of the FRAILMar cohort. The prevalence of malnutrition was high (34.9%), within the range from 11 to 54% as previously reported in studies of protein energy wasting [36,37] and from 50% to 75% in hemodialysis patients [38].

The ESPEN guidance for the assessment of the GLIM muscle mass phenotypic criterion highlights the PhA as a topic that deserves further research [12]. The association of PhA values with malnutrition has been reported, but the majority of these studies used the SGA as the reference standard instead of the GLIM criteria and were conducted mainly in older populations [12]. To the authors’ knowledge, only one study (on 70 hemodialysis patients from the United Arab Emirates) has assessed the clinical usefulness of PhA applying the GLIM criteria [38], reporting a higher prevalence of malnutrition (54.3%) and similar mean values of PhA (4.66° (SD 1.21)) compared to the present study. They applied a cutoff point of 5.7° with good sensitivity (86.8%) but low specificity (35.5%) and assessed muscle mass with the fat-free mass index, using <15 (women) and <17 kg/m^2^ (men) as the cutoff points, based on Swiss reference material [17].

Some limitations of the study should be acknowledged. First, the sample size was relatively small. Second, our study did not have access to data about how long patients had been receiving renal replacement therapy, which could have been a variable of interest. Third, a potential for selection bias existed, as participants in the FRAILMar study were candidates for KT and therefore patients with severe CKD were not included if clinical characteristics (i.e., severe disability or advanced dementia) prevented KT candidacy. As a result, the findings cannot be applied to this population. Fourth, the timing of the assessment is a potential limitation. Although very few studies have rigorously evaluated the optimal timing for the assessment of body composition in CKD, the best timing appears to be when patients are close to their dry weight or when patients undergoing peritoneal dialysis have an empty cavity [7]. Only 18 patients in our sample had a normal hydration state (44% of hemodialysis patients, 25% of peritoneal dialysis patients, and 26.6% of non-dialysis patients). Although patients on peritoneal dialysis were told to attend with an empty cavity, this condition was not always achieved because many patients had lengthy travel times to the hospital where assessments were conducted. This is particularly relevant for methods that cannot distinguish between ICW and ECW (e.g., DXA), but is not the case for the BIA device used in this study (InBody S10®). Bioimpedance analysis, in particular BIS, is the current preferred method for assessments of body composition in patients with end-stage renal disease [39]. Although BIS and BIA may seem to be the same, they have slightly different features. Multifrequency BIA methods (e.g., InBody S10®) and BIS use the same range of frequencies (0 to 1000 Hz), the InBody S10 device uses six frequencies, and spectroscopy systems use a greater number of frequencies, which might deliver better precision. Another important difference is that BIS fluid estimates do not depend on empirical equations from a given population, since the fluid compartment is estimated using the Cole–Cole model [40]. This model takes into account the fat content, which is inversely related to fluids (TBW, ICW, and ECW) [41]. Our study used the InBody S10® method, which—in contrast to other BIA devices—estimates fluids using the Cole–Cole method, as does the BIS approach. This feature makes it an excellent BIA device to assess body composition in patients with advanced CKD. Finally, we must bear in mind that the association between PhA and sarcopenia was not explored. Sarcopenia is a reality in patients with CKD, especially in patients on a hemodialysis program [13], and further research is certainly needed.

In summary, BIA results are highly influenced by body water and hydration status, and changes in body water result in underestimation (in the case of dehydration) or overestimation (overhydration and edema) of muscle mass; this is particularly important in patients with advanced CKD. The PhA, a calculation based on reactance (Xc) and resistance (R), measures how long the signal is delayed by Xc [32]. Reduced PhA values are associated with cell dysfunction, inflammation, nutritional disorders, and mortality [42,43,44,45,46]. Although in theory the PhA measurement is not affected by ECW, patients with high ECW have lower PhA values; a possible explanation for this is that the loss in muscle mass involves a decrease in ICW, decreasing both the R and PhA values [47]. In our study, the performance properties of PhA were improved in the subsample of patients with a BMI <25 kg/m^2^, suggesting that when BIA availably is limited, the measurement of PhA may provide valuable clinical and prognostic information on these patients. The measurement of PhA is a simple, non-invasive, and low-cost follow-up that can easily be available in rehabilitation units. These issues deserve further study to explore the applicability of serial PhA measurements in the nutritional assessment of patients with advanced CKD.

## 5. Conclusions

Malnutrition according to the GLIM criteria was frequent (34.9%) in patients with advanced CKD on the waiting list for KT in the baseline assessment of the FRAILMar study. Most of these patients had PhA values ≤ 4.85°, which was also the PhA threshold yielding the highest accuracy (sensitivity 72.7%, specificity 65.9%, positive and negative likelihoods ratios 2.13 and 0.41, respectively; i.e., fair validity). The performance properties of the PhA improved in the subsample of participants with a BMI < 25 kg/m^2^. Overall, the PhA values ≤ 4.85° were associated with a 3.8-fold higher malnutrition risk (OR = 3.8 (CI95% 1.1 to 13.8)). Considering the GLIM criteria as the reference standard, performance testing of PhA ≤ 4.85° showed only fair validity for identifying patients with and without malnutrition in this preliminary analysis. Pending further studies, it cannot be recommended as a stand-alone screening tool for malnutrition in KT candidates.

## Figures and Tables

**Table 1:** Demographic and clinical characteristics of the FRAILMar study participants.

	Total Sample (*n* = 63)	Range of Normality
Age (years)	62.9 (SD 10.9)	-
Sex, men (%)	48 (76.2%)	-
Dialysis modality, n (%)		
Hemodialysis	36 (57.1%)	
Peritoneal dialysis	12 (19.0%)	-
No renal replacement therapy	15 (23.8%)	
Body mass index (kg/m^2^)	28.4 (SD 5.1)	18.5–25 kg/m2 [33]
Frailty, Fried phenotype 3–5 (%)	26 (41.3%)	
Malnutrition, GLIM criteria (%)	22 (34.9%)	
BIA-derived parameters:		
Appendicular musculoskeletal mass index (kg/m^2^) ^a^	7.7 (SD 1.2)	-
Musculoskeletal mass (kg) ^a^	27.7 (SD 5.4)	-
Fat-free mass (kg) ^a^	51.1 (SD 9.2)	-
Fat-free mass (% ref.)	95.2 (SD 13.6)	90–110% [17]
Fat mass (kg) ^a^	27.4 (SD 12.1)	-
Fat mass (% body weight)	33.8 (SD 10.1)	Men 10–20%, women 18–28% [34]
Total body water (L) ^b^	37.8 (SD 6.9)	-
Extracellular water (L)	15.0 (SD 2.8)	-
Intracellular water (L)	22.8 (SD 4.1)	-
Extracellular water/total body water	0.397 (SD 0.011)	0.360–0.390 [18,19]
Phase angle (°)	5.0 (SD 0.9)	5–7° [9]
Muscle strength of the dominant side:		
Handgrip strength (kg) ^a^	28.4 (SD 8.2)	-
Handgrip strength (%ref.)	80.8 (SD 19.4)	80–120% [22]
Muscle size assessed using ultrasound:		
Muscle thickness of dominant forearm (mm) ^b^	15.1 (SD 3.9)	13.3–23.5 mm [23]
Muscle thickness of dominant *rectus femoris* (mm) ^b^	17.2 (SD 4.3)	Men 20–31 mm; women 16–24 mm [35]

^a^ Values depending on age and sex. ^b^ Values depending on individual characteristics.

**Table 2:** Contingency tables showing the frequency distribution of the phase angle values (cutoff point ≤4.85°) in candidates on the waiting list for kidney transplantation (A) and in the subgroup of patients with body mass index <25 kg/m^2^ (B).

A	Reference Standard (GLIM Criteria)			
		Malnutrition (*n* = 22)	No Malnutrition (*n* = 41)	Total Sample (*n* = 63)
Phase angle ≤ 4.85°	Positive	16	14	30
	Negative	6	27	33
**B**		**Malnutrition** **(*n* = 11)**	**No Malnutrition** **(*n* = 9)**	**Patients with** **BMI < 25 kg/m^2^ (*n* = 18)**
Phase angle ≤ 4.85°	Positive	9	2	11
	Negative	2	7	9

**Table 3:** Performance properties of the index test (reduced phase angle) to detect malnutrition according to the Global Leadership Initiative on Malnutrition (GLIM) criteria in patients with advanced chronic kidney disease on the waiting list for kidney transplantation (A), and in the subgroup of patients with body mass index <25 kg/m^2^ (B).

	All of the Sample	In the Subgroup of Patients with BMI < 25 kg/m^2^
Sensitivity	72.7%	81.8%
Specificity	65.9%	77.8%
Positive predictive value	53.3%	81.8%
Negative predictive value	81.8%	77.8%
Accuracy	68.3%	80%
Positive likelihood ratio	2.13	3.68
Negative likelihood ratio	0.41	0.23

**Abbreviations:** BMI: body mass index.

**Table 4:** Univariate analysis comparing patients according to values of the phase angle (≤4.85°).

	Phase Angle ≤ 4.85° (*n* = 30)	Phase Angle > 4.85° (*n* = 33)	Mean Differences (95% CI)	*p*-Value
Age (years)	66.5 (SD 7.6)	59.6 (SD 12.4)	6.9 (1.7 to 12.0)	0.010
Sex, men (%)	23 (76.7%)	25 (75.8%)	-	0.933
Dialysis modality, n (%)				
Hemodialysis	19 (63.3%)	17 (56.7%)		
Peritoneal dialysis	6 (20%)	6 (20%)	-	0.441
No renal replacement therapy	5 (16.7%)	10 (33.3%)		
Body mass index (kg/m^2^)	28.3 (SD 5.3)	28.4 (SD 5.1)	−0.17 (−2.8 to 2.4)	0.898
Frailty, Fried phenotype 3–5 (%)	17 (56.7%)	9 (27.3%)	-	0.018
Malnutrition, GLIM (%)	16 (53.3%)	6 (18.2%)	-	0.003
BIA-derived parameters:				
Appendicular musculoskeletal mass index (kg/m^2^)	7.4 (SD 1.3)	8.0 (SD 1.0)	−0.6 (−1.2 to 0.01)	0.057
Musculoskeletal mass (kg)	26.4 (SD 5.9)	28.9 (SD 4.6)	−2.4 (−5.1 to 0.24)	0.074
Fat-free mass (kg)	49.5 (SD 10.2)	52.7 (SD 7.9)	−3.2 (−7.8 to 1.4)	0.169
Fat-free mass (% ref.)	92.0 (SD 14.4)	98.1 (SD 12.3)	−6.1 (−12.8 to 0.7)	0.076
Fat mass (kg)	28.9 (SD 12.1)	26.0 (SD 11.6)	2.9 (−3.2 to 9.0)	0.351
Fat mass (% ref.)	35.9 (SD 10.1)	31.9 (SD 9.9)	4.0 (−1.1 to 9.0)	0.119
Total body water (L)	36.6 (SD 7.7)	38.8 (SD 5.9)	−2.2 (−5,7 to 1.2)	0.203
Extracellular water (L)	14.8 (SD 3.2)	15.2 (SD 2.4)	−0.368 (−1.788 to 1.051)	0.606
Extracellular water/Total body water	0.404 (SD 0.009)	0.390 (SD 0.008)	0.014 (−0.009 to 0.018)	<0.001
Muscle strength of dominant side:				
Handgrip strength (kg)	26.0 (SD 7.6)	30.6 (SD 8.2)	−4.6 (−8.6 to 0.6)	0.025
Handgrip strength (% ref.)	77.0 (SD 19.6)	84.3 (SD 18.7)	−7.3 (−16.9 to 2.4)	0.137
Muscle size assessed using ultrasound:				
Muscle thickness of dominant forearm (mm)	14.0 (SD 3.6)	16.2 (SD 3.8)	−2.2 (−4.1 to −0.3)	0.021
Muscle thickness of dominant *rectus femoris* (mm)	15.3 (SD 3.9)	19.0 (SD 4.0)	−3.7 (−5.7 to −1.7)	<0.001

**Note:** Student *t*-test for independent samples was used for comparisons of quantitative variables; chi-square test was used for categorical variables.

**Table 5:** *Odds ratios* of malnutrition in patients with phase angle ≤4.85° (dependent variable) by frailty, age, and handgrip. Crude analysis and adjusted results.

	Crude Analysis (Univariate)			Multivariate Analysis		
**Malnutrition**	**cOR**	**95%** **CI**	***p***	**aOR**	**95%** **CI**	***p***
Phase angle (≤4.85°)	5.14	1.7 to 16.1	0.005	3.8	1.05 to 13.8	0.042
Frailty				3.7	0.96 to 14.3	0.058
Age				1.0	0.98 to 11.6	0.856
Handgrip (kg)				1.0	0.9 to 1.1	0.561

**Abbreviations:** % ref.: percentage of population reference value; cOR: crude odds ratio; aOR: adjusted odds ratio.

## Data Availability

Further supporting data are available from the authors upon request.
